# Case Report: endoscopic thyroidectomy via total areola approach in a six-year-old patient with thyroid follicular adenoma

**DOI:** 10.3389/fped.2025.1549049

**Published:** 2025-04-29

**Authors:** Qingqing Cai, Yifan Ke, Wenchao Li, Peng Zhang, Jiezhong Wu, Kunpeng Hu

**Affiliations:** Department of Breast and Thyroid Surgery, Lingnan Hospital, The Third Affiliated Hospital of Sun Yat-sen University, Guangzhou, China

**Keywords:** thyroidectomy, endoscopic surgical procedures, areola, child, age factors, thyroid nodule, adenoma, follicular

## Abstract

**Background:**

Endoscopic thyroidectomy (ET) has become increasingly popular globally, but its application in young children remains largely unexplored. This study reports a 6-year-old girl with a neck mass who underwent ET via total areola approach.

**Methods:**

After ultrasonographic (ACR TI-RADS 4) and cytological (TBSRTC 4) confirmation, the patient underwent endoscopic right and isthmic thyroidectomy with inferior parathyroid auto-transplantation under general anesthesia. The procedure utilized 3 mm pediatric instruments, intraoperative neuromonitoring and near-infrared auto fluorescent parathyroid monitoring.

**Results:**

The procedure achieved complete resection with 215 min operative time and minimal blood loss. The pathological diagnosis was thyroid follicular adenoma. At 3-month follow-up, no complications and excellent cosmetic outcomes were observed.

**Conclusion:**

ET via total areola approach proves highly suitable for young children because of its safety and cosmetic advantages. Experienced surgeons, small-size special surgical instruments and auxiliary monitoring techniques are helpful to improve the safety of pediatric ET.

## 1 Introduction

Thyroidectomy is one of the main treatments for thyroid diseases in children. In recent years, the number of pediatric patients requiring thyroidectomy has been increasing. Currently, the indications, techniques and complications of thyroidectomy in pediatric patients are similar to those in adults (1).

Thyroidectomy includes open thyroidectomy (OT), which is performed through a neck incision, and endoscopic thyroidectomy (ET), which is performed through concealed incisions in the areola, axilla, mouth floor, and post aurem. Compared to ET, the main disadvantage of OT is visible neck scarring. For pediatric patients, especially female, these scars are more likely to impair their mental health and quality of life. Besides, ET possesses other advantages such as high-definition visual field and less hemorrhage during operation.

At present, the safety and efficacy of thyroidectomy in pediatric patients is acceptable, but the operation volume is far less than that in adults. Therefore, we need more cases for clinical reference. This paper describes a 6-year-old girl with thyroid follicular adenoma who underwent ET via the total areola approach. According to our search, she is the youngest patient to have undergone this approach to date.

## 2 Case description

### 2.1 Patient

On July 30, 2024, a 6-year-old girl was hospitalized in the Department of Thyroid and Breast Surgery in our center for a neck mass found more than 2 years ago without any symptoms such as pain and fever. She had a palpable, oval, soft-textured, well-defined, non-tender mass measuring approximately 2*1.5 cm in diameter in the right anterior cervical triangle. No treatment had been conducted prior to this operation. Results of preoperative examinations such as thyroid function, parathyroid function, electrocardiogram, and fiberoptic laryngoscopy were normal. Ultrasonography revealed a thyroid nodule in the right lobe, classified as ACRTI-RADS Grade 4. Fine-needle aspiration biopsy suggested that it might be a follicular tumor with TBSRTC Grade 4. There is no family history of genetic diseases or mental disorders.

### 2.2 Surgical techniques

**Figure 1:**
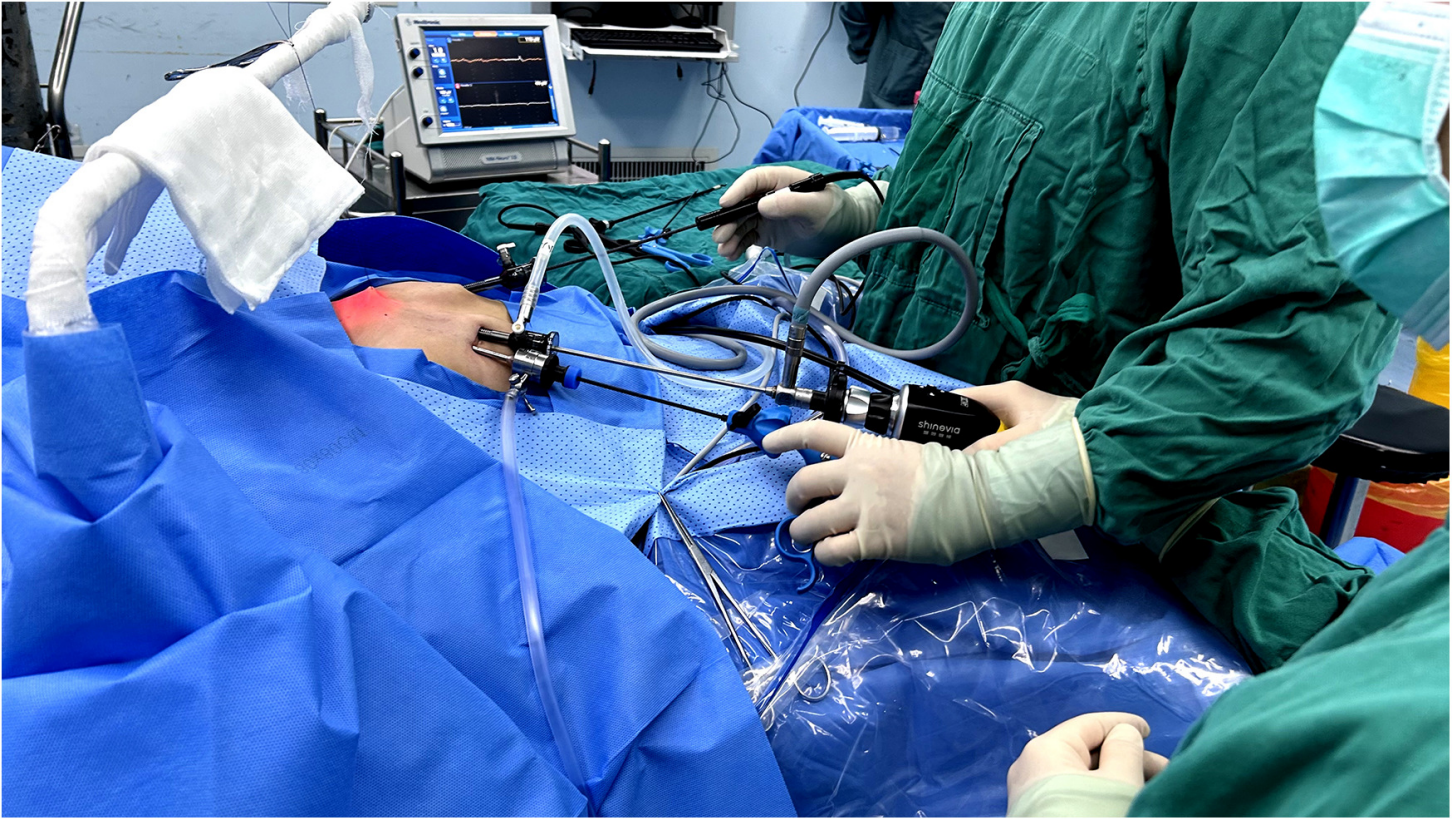
A photo of the intraoperative trocar position.

**Figure 2:**
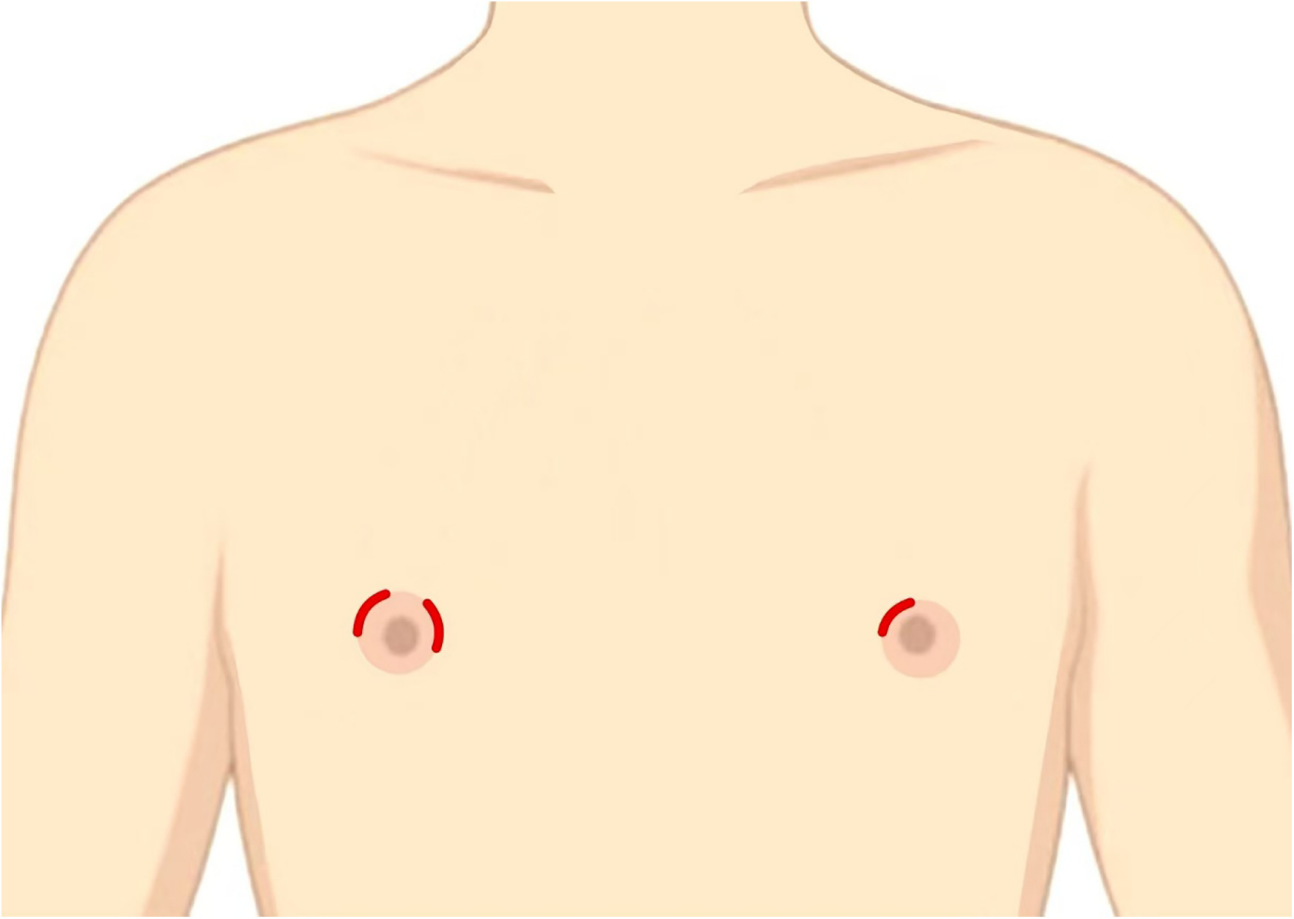
Intraoperative trocar set-up.

The patient underwent endoscopic right and isthmic thyroidectomy with inferior parathyroid auto-transplantation under general anesthesia on August 1, 2024. The patient was placed in the supine split-leg position, and the laparoscopic system, the ultrasonic scalpel, the suction pump, and intraoperative neuromonitoring (IONM) device was connected. The surgeons made curved incisions with a length of 5–8 mm respectively in the 11 o'clock direction of the bilateral areola and in the 3 o'clock direction of the right areola (Figures 1, 2). Then they chose the 3 mm and 5 mm diameter trocars, which were more suitable for pediatric patients. The subcutaneous tissue was separated up to the anterior neck region with a 3 mm grasping forceps and the ultrasonic scalpel, and the linea alba was cut layer by layer to expose the right lobe and the lesion. The thyroid capsule was carefully dissected to identify recurrent laryngeal nerve (RLN) and parathyroids. Two parathyroids were located and confirmed by a near-infrared auto fluorescent (NIRAF) detector. The superior parathyroid was preserved, while the inferior parathyroid with insufficient blood supply was transplanted into the right sternocleidomastoid muscle. The RLN and vagus nerve were detected by the IONM, which determines the location and conduction of nerves by stimulating and receiving the electrical signals. Eventually, the right thyroid lobe, isthmus, and lesion was completely resected and pulled out with a specimen bag. The surgical field was thoroughly stanched and irrigated, and a drainage tube was placed.

### 2.3 Results

**Figure 3:**
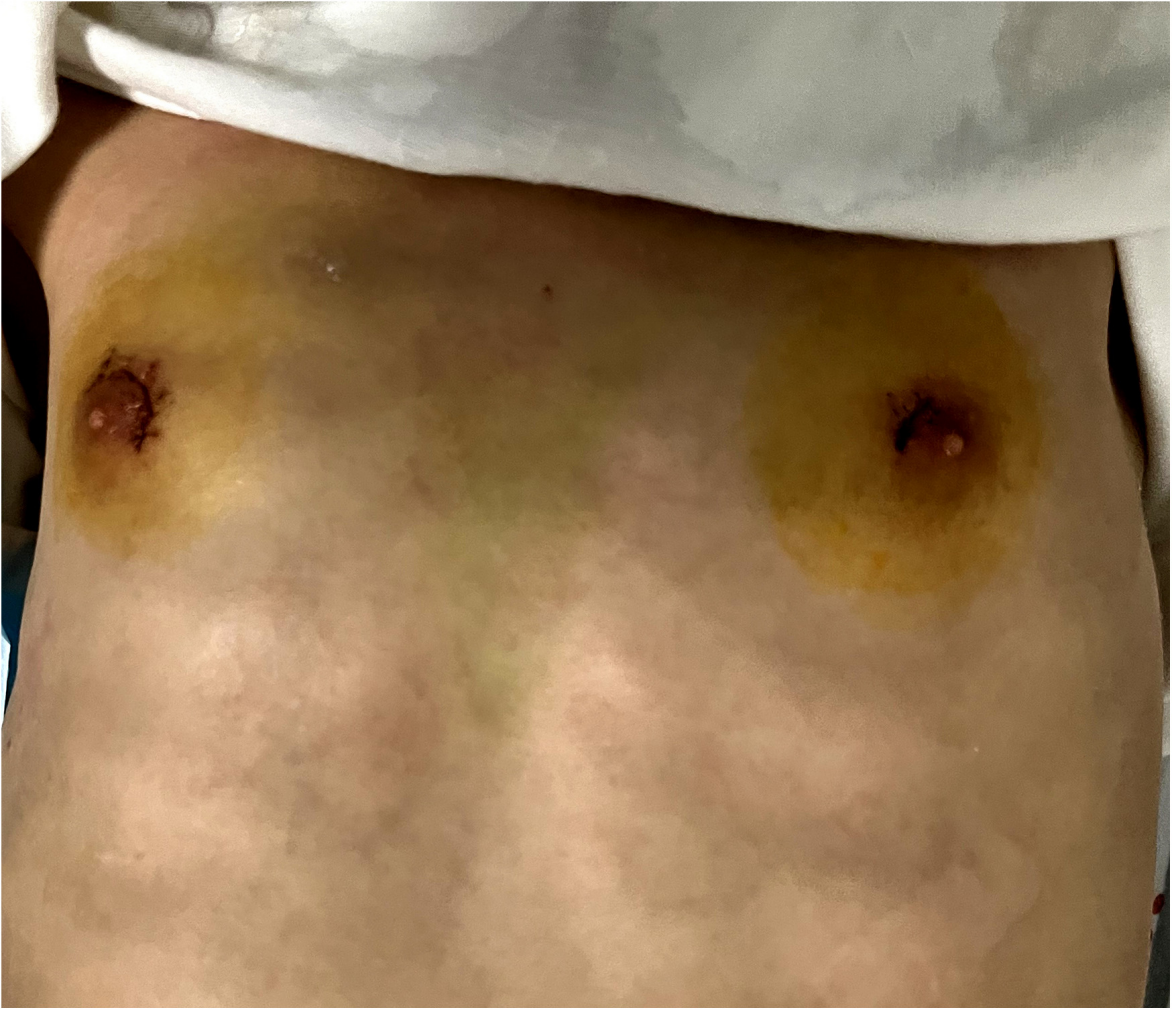
The incisions one day after operation.

The operation was successfully finished without conversion to OT and the blood loss was about 20 ml. The operative time was 215 min. After the operation, the patient reported mild pain in the neck and fully cooperated with the postoperative treatments which included electrocardiographic monitoring, low-flow oxygen therapy, analgesia, nebulization, intravenous fluids and calcium supplementation. On the 2nd postoperative day, the patient was able to ambulate and received a liquid diet and oral levothyroxine sodium. Within 3 days after surgery, the drainage tube discharged 35 ml, 10 ml, and 8 ml of light red fluid, respectively. On the 3rd postoperative day, the drainage tube was removed and the patient was discharged without any complication. The surgical incisions healed well without local infection or dehiscence (Figure 3). And the diagnosis of thyroid follicular adenoma was confirmed by the pathological result. The patient was followed up for 3 months after discharge, without any surgical complications occurred. Re-examination of neck ultrasonography and thyroid function showed no abnormality. The patient and her family members expressed their contentment with both the surgical efficacy and the post-operative cosmetic outcome.

## 3 Discussion

During the operation, we found that the parathyroids were more difficult to recognize in children. And the cervical region VI was covered by the larger thymus. Besides, the volume of thyroid is age dependent, the average volume of 7-year-old children is less than 4 cm^3^ (2), which is much smaller than that of adults, and the same is true for the vessels and parathyroid. As a result, the risk of complications for thyroidectomy is higher in children. Existing studies have shown that the incidence of postoperative permanent hypocalcemia in adults and pediatric patients is 0.87% (3) and 1.16% (1), respectively. In addition, the RLN palsy, wound infection, keloid formation, hemorrhage, and chylous leakage are also complications of thyroidectomy in children. Therefore, it is necessary for surgeons to pay more attention to the protection of parathyroid glands, nerves and blood vessels, especially for children aged 0–6 years.

The application of ET provides a good solution to this issue. The endoscope has a high-definition field of view and the ability to zoom, which allows close-up presentation of important anatomical structures within the surgical field. During the ET in pediatric patients, surgeons can distinguish the small-size parathyroids and blood vessels and even the smaller structures which were difficult to achieve with naked eye. In addition, endoscopic modified instruments, such as 3 mm grasping forceps, improve the delicacy of the surgeon's manipulation, making ET in children safer and more efficient. Due to the unique anatomical and physiological features, thyroidectomy in pediatric patients requires more expertise and experience than in adults. A study revealed that children treated by higher-volume surgeons (more than 30 cases per year) tend to have fewer complications following thyroidectomy (4). As the thyroid gland is highly vascularized, meticulous hemostasis is essential to ensure surgical safety. As demonstrated by Sengul et al. (5) and Aydin et al. (6), the combined use of energy-based devices and IONM in thyroidectomy significantly facilitates surgical procedures, reduces blood loss, and enhances recurrent RLN preservation. This technical combination proves particularly advantageous for pediatric cases, as exemplified by our 6-year-old patient. Furthermore, Benmiloud et al. (7) established that near-infrared autofluorescence (NIRAF) detection improves parathyroid identification accuracy and reduces postoperative transient hypocalcemia rates from 21.7% to 9.1%. In the present case, we achieved optimal outcomes through conducting hemostasis and dissection with ultrasonic scalpel complemented by IONM and NIRAF, resulting in minimal blood loss (10 ml) and no incidence of RLN injury or hypocalcemia during the 3-month follow-up period. In summary, we recommend that high-volume surgeons perform pediatric ET with the assistance of small-sized modified instruments, IONM, and NIRAF detectors to further reduce the incidence of surgical complications.

At present, there are far fewer reports of ET in pediatric patients than in adults. A retrospective clinical study evaluated 31 children who underwent the trans-axillary endoscopic head and neck endocrine surgery, but 8 of them suffered transient minor complications (2). The other 2 case reports described pediatric patients who underwent trans-oral ET (8, 9). However, these papers did not emphasize differences between pediatric and adult ET and offer insights on how to further reduce the incidence of surgical complications.

Another advantage of ET is the cosmetic effect of a scarless neck. Children with head or neck scars are at increased risk of depression and the scars may adversely affect childhood psychosocial functioning (10). Scars resulting from OT can be noticeable and have a significant impact on one's day-to-day routines and emotion (11). The neck scar following OT was found to be associated with a lower quality of life in the pediatric patients (12). A questionnaire survey revealed that psychological distress immediately after operation and during surveillance period was higher in OT than in bilateral axillo-breast approach robotic thyroidectomy (13). In our case, the patient demonstrated high satisfaction with the scarless outcome during the 3-month follow-up. This further supports the cosmetic value of ET in pediatric populations with increasing thyroid diseases.

Among all ET approaches, the total areola approach has several advantages as follows. Firstly, the natural pigmentation of the areola helps to conceal the incisions, resulting in better cosmetic results. Second, compared to the trans-oral approach, the total areola approach takes a lower risk of infection due to its class I incision and provides a more intuitive and classic surgical perspective. Third, compared to the trans-axillary approach and the post-aurem approach, the total areola approach takes a lower risk of serious complications such as neurovascular injury. In addition, compared to robotic thyroidectomy, ET has a shorter learning curve, lower cost, and shorter operation time (14). To sum up, our experience suggests that ET via total areola approach may be a highly suitable option for pediatric patients with thyroid diseases. This paper is a useful reference for selecting surgical approaches for pediatric thyroidectomy.

This case provides evidence supporting the safety and feasibility of endoscopic resection for benign thyroid tumors. However, the applicability of ET in pediatric malignant thyroid disease warrants cautious consideration. Recent data indicate that the malignancy rate of thyroid nodules in children is higher than 60% (15), necessitating rigorous evaluation of ET's role in malignant cases. For patients with malignancy confined to the thyroid without radiologically evident lymph node metastasis, total thyroidectomy with central lymph node dissection (CND) remains standard. The study by Jiang et al. demonstrated that the tumor recurrence rates and the level of surgical completeness in ET are comparable to those in OT (16). However, pediatric-specific long-term follow-up data on recurrence and survival rates are still lacking. Notably, the areolar approach provides adequate exposure for CND in our experience, as the endoscopic magnification facilitates meticulous dissection of central compartment structures. For patients requiring lateral neck dissection due to radiologically confirmed lymph node metastasis, ET's limitations become apparent. The restricted operative field and technical challenges in accessing lateral nodal stations may compromise oncologic completeness. This highlights the need for individualized surgical planning and multidisciplinary evaluation when malignancy is suspected.

While ET offers numerous advantages, this approach is not without significant limitations and shortcomings. Initially, the prolonged operative time must be acknowledged. As evidenced by Cao et al. in their comparative study of benign thyroid tumors, ET required substantially longer operative durations for both unilateral lobectomy (79.9 ± 20.10 vs. 45.4 ± 11.90 min, *P* < 0.001) and bilateral total thyroidectomy (89.9 ± 14.60 vs. 60.0 ± 8.44 min, *P* < 0.001) (17). This temporal disparity primarily stems from additional subcutaneous tunnel creation time and the technical constraints of operating within a restricted visual field. Importantly, these temporal demands may be further exacerbated in pediatric populations due to their characteristically reduced anatomical working space. Moreover, ET demands greater surgical expertise and incurs higher economic costs. Furthermore, ET carries unique procedure-related complications, as illustrated by Lobe et al.'s pediatric series where 1 of 31 cases (3.2%) developed postoperative subcutaneous hematoma (18). Other potential endoscopic-specific complications include subcutaneous tunnel paresthesia, flap perforation or necrosis, tumor implantation or recurrence around subcutaneous tunnel and so on.

## 4 Conclusion

ET via total areola approach demonstrates safety and feasibility in pediatric patients, offering advantages over OT through reduced surgical complications and superior cosmetic outcomes, minimizing physical and psychological trauma. Furthermore, ET performed by experienced surgeons utilizing pediatric-specific miniature instruments and auxiliary monitoring techniques can further improve its efficacy and safety. Further multicenter studies are warranted to validate these findings and refine technical protocols.

## Data Availability

The datasets presented in this article are not readily available because. The dataset cannot be shared with third parties without prior written consent. Any modification to the dataset is strictly prohibited without proper authorization. Requests to access the datasets should be directed to Kunpeng Hu, hukpeng@mail.sysu.edu.cn.
